# Supplementary material for: The role of noninfectious comorbidities in the association between severe infections and risk of dementia in Finland: A nationwide registry study
Source: PLoS Med. 2026 Mar 24;23(3):e1004688. doi: 10.1371/journal.pmed.1004688 (PMC13012496; doi:10.1371/journal.pmed.1004688)
Supplement: S1 Study Plan — (PDF) [file pmed.1004688.s001.pdf]

Research plan

Pyry Sipilä

19.7.2022

Project title: "Socioeconomic status, infections, and disease trajectories leading to dementia"

Summary

Dementia is the fifth leading cause of death worldwide.(1) It is accompanied by inflammation in the brain and many studies have found infectious diseases to be associated with an increased risk of dementia.(2–6) However, it is unclear whether infections have a causal role in the development of dementia at the early stages of neurodegeneration (the causal hypothesis) or whether infections are only markers of underlying socioeconomic disadvantage, poor health, and multimorbidity, which may be accompanied by cognitive decline and neurodegeneration (the marker hypothesis). To increase understanding of the role of socioeconomic factors and infectious and other diseases in the aetiology of dementia, this project will characterise the temporal order and trajectories of infectious and other diseases preceding dementia. The specific aim is:

AIM: To analyse trajectories of infectious and other diseases preceding dementia and the role of socioeconomic factors in these trajectories

Study question: Do infections often occur early in the disease trajectories leading to dementia (supporting the causal hypothesis) or do the disease trajectories leading to dementia typically include several other diseases before infections start to develop (supporting the marker hypothesis)? How do the association between disease trajectories, infections and dementia vary by socioeconomic position?

The project will use total population data from Finland. The results will clarify the role of socioeconomic status and infectious and other diseases in the aetiology of dementia and inform the prevention of Alzheimer's disease and other dementias.

Outline of the study and its methods

Setting and outcome definitions

We use data from 1.1.1996 (the start of ICD-10 records in Finland) until present (the latest update of the records).

We pick all dementia cases in inpatient or outpatient hospital records (ICD-codes F00, F01, FO2, F03, F05.1, G30, G31.0, G31.1, G31.8) and medication reimbursement entitlements for the treatment of dementia (Alzheimer's disease and Parkinson's disease dementia) between 1.1.2017 and present. We exclude those with pre-existing dementia records before 1.1.2017, either in hospital or in medication reimbursement records. We also exclude those who were <40 years old at dementia diagnosis.

-Note: Unfortunately, from 1.11.2016 onwards, medication reimbursement entitlements were granted for rivastigmine only, but I would still suggest including these records.

For each dementia case, we pick 5 controls free of dementia on the day of dementia diagnosis of the case, matched for year of birth sex, area of residence, education, and marital status. We use incidence density sampling so that the controls can become cases if their develop dementia after they have been selected as controls.(7)

We pick covariates 21 years before dementia diagnosis, starting from 1.1.1996 (or closest available date to that if information on that day is not available), because exposure recording starts on that day (please see below).

-We classify education “as the highest achieved qualification, categorised as tertiary (generally ≥13 years of education), secondary (10–12 years) and basic education or less (up to 9 years)” (as in Korhonen et al.)(8)

-We classify marital status as “married, divorced, widowed, and never married” (as in Korhonen et al.)(8)

-For area of residence, we choose a reasonable classification that is available (e.g., health care district [sairaanhoitopiiri]).

Exposures

We pick hospitalisations that happened 1 to 21 years before the index date (date of dementia for cases, the same date for controls). This provides a 20-year span with a 1-year gap between the latest hospitalisations and dementia diagnosis. The gap is used, because hospitalisations that occur shortly before dementia diagnosis are highly likely to be affected by the developing dementia. We use 3-digit ICD-10 codes from primary diagnoses.

Preliminary structure of the manuscript and its analyses

The methodology described below is adapted from that used in Kivimäki et al.(9)

Figure 1

Flowchart of the selection of participants

Table 1

Basic characteristics of the participants (one column for cases and another for controls)

Figure 2

List of the 100 most common ICD-10 codes (on 3-digit level) for primary reasons of hospitalisations that precede the diagnosis of dementia (during the 20-year timeframe described above). We show N (%) for each diagnosis among cases and controls and the respective odds ratio (OR) with 95% confidence interval and Bonferroni-corrected p-value. We also show N (%), OR (95% CI) and corrected p-value for no hospitalisation during that timeframe.

-For further analysis, we pick those conditions which are reasonably common (≥1% of those with dementia have a history of the diagnosis) and are more common among those with dementia than among controls (OR ≥1.5 with corrected p-value <0.05 as a threshold). If this yields an unpractical number of diseases, the thresholds (1% and OR 1.5) may be adjusted.

Figure 3

From diseases retained in analysis, we form disease pairs, and for each pair, we check which one tends to occur earlier among those dementia cases who had both of the diseases. We compute hazard ratios for the second disease of each pair among all dementia cases who had the first disease. The hazard ratios will be adjusted for age and the matching variables (sex, education, marital status, area of residence).

Figure 4

From diseases with a statistically significant HR of ≥ 2, we construct disease trajectories that lead to dementia (please see below an example figure from Kivimäki et al.)(9) If this yields an unpractical number of diseases, the HR threshold may be adjusted. If there are any diseases that are significantly associated with dementia (OR≥1.5) but are not included in any trajectories, we add them separately to Figure 3. The x-axis in Figure 3 is the average age of hospitalisation (for diseases preceding dementia) or diagnosis (for dementia).

Example of Figure 4 (the figure is from ref. 9 and is under the CC BY 4.0 license, https://creativecommons.org/licenses/by/4.0/)

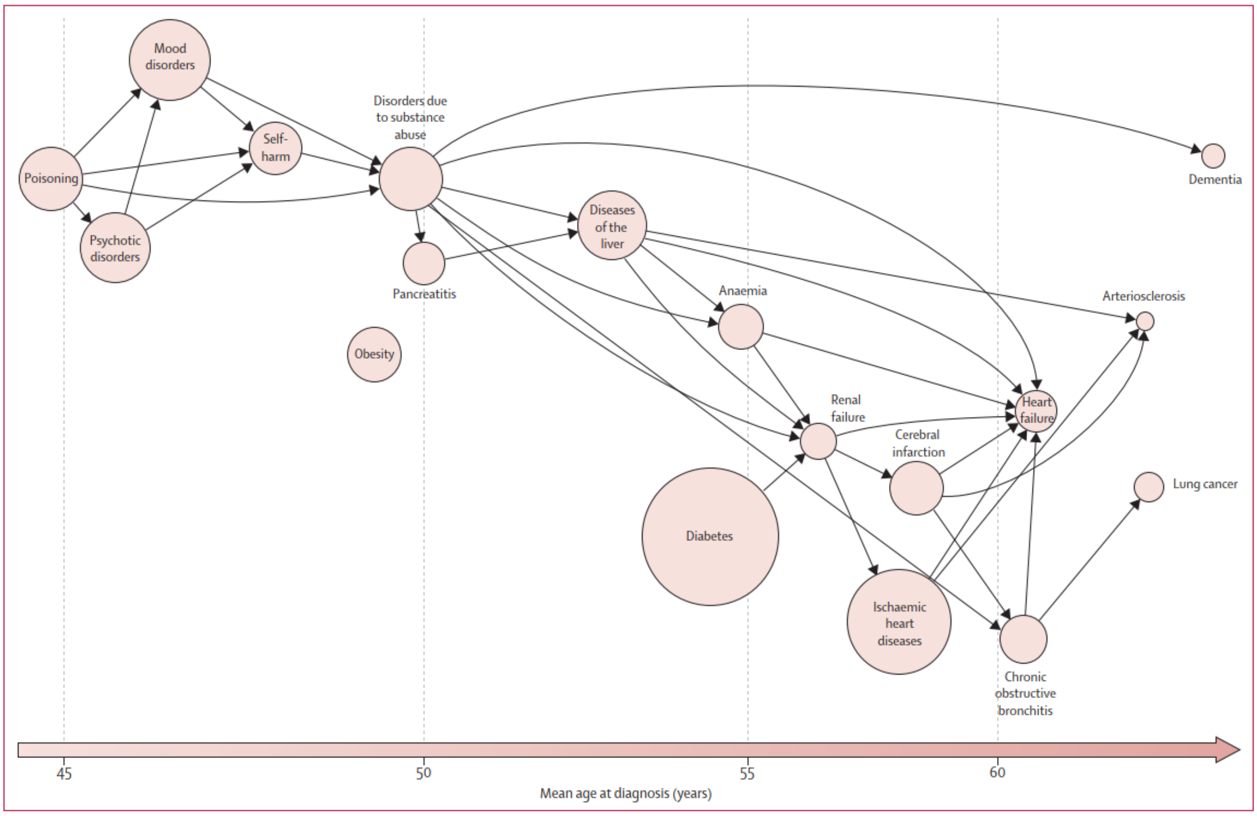

Figure 5

Repeating the methodology above, we construct Figure 5 that is like Figure 4 but divided to 3 panels, one for each level of education. We also construct eFigure1 and eFigure2 that are like Figures 2 and Figure 3 but stratified by education. For analyses by educational level, we use the same thresholds as in the main analysis.

References

1. Nichols E, Szoeke CEI, Vollset SE, Abbasi N, Abd-Allah F, Abdela J, et al. Global, regional, and national burden of Alzheimer's disease and other dementias, 1990–2016: a systematic analysis for the Global Burden of Disease Study 2016. Lancet Neurol. 2019 Jan;18(1):88–106.

2. Muzambi R, Bhaskaran K, Smeeth L, Brayne C, Chaturvedi N, Warren-Gash C. Assessment of common infections and incident dementia using UK primary and secondary care data: a historical cohort study. Lancet Healthy Longev. 2021 Jul;2(7):e426–35.

3. Muzambi R, Bhaskaran K, Brayne C, Davidson JA, Smeeth L, Warren-Gash C. Common Bacterial Infections and Risk of Dementia or Cognitive Decline: A Systematic Review. J Alzheimers Dis. 2020;76(4):1609–26.

4. Shi Y, Holtzman DM. Interplay between innate immunity and Alzheimer disease: APOE and TREM2 in the spotlight. Nat Rev Immunol. 2018 Dec;18(12):759–72.

5. Sipilä PN, Heikkilä N, Lindbohm JV, Hakulinen C, Vahtera J, Elovainio M, et al. Hospital-treated infectious diseases and the risk of dementia: a large, multicohort, observational study with a replication cohort. Lancet Infect Dis. 2021 Nov;21(11):1557–67.

6. Sipilä PN, Lindbohm JV, Singh-Manoux A, Shipley MJ, Kiiskinen T, Havulinna AS, et al. Long-term risk of dementia following hospitalization due to physical diseases: A multicohort study. Alzheimers Dement. 2020;16:1686–95.

7. Rothman KJ, Lash TL, VanderWeele TJ, Haneuse S. Modern epidemiology. Fourth edition. Philadelphia: Wolters Kluwer; 2021.

8. Korhonen K, Tarkiainen L, Leinonen T, Einiö E, Martikainen P. Association between a history of clinical depression and dementia, and the role of sociodemographic factors: population-based cohort study. Br J Psychiatry. 2022 Jul;221(1):410–6.

9. Kivimäki M, Batty GD, Pentti J, Shipley MJ, Sipilä PN, Nyberg ST, et al. Association between socioeconomic status and the development of mental and physical health conditions in adulthood: a multi-cohort study. Lancet Public Health. 2020 Mar;5(3):e140–9.
