# Supplementary material for: The role of noninfectious comorbidities in the association between severe infections and risk of dementia in Finland: A nationwide registry study
Source: PLoS Med. 2026 Mar 24;23(3):e1004688. doi: 10.1371/journal.pmed.1004688 (PMC13012496; doi:10.1371/journal.pmed.1004688)
Supplement: Attachment — Submitted filename: Sipila et al. Response_to_reviewers_R2.docx [file pmed.1004688.s007.docx]

**Comments from the reviewers:**

**RESPONSE TO REVIEWER COMMENTS – REVIEWER #1:**

**POINT:**

**Reviewer #1: "Severe infections and risk of dementia in Finland: The role of comorbidities in a nationwide prospective study" attempts to disentangle the effects of other comorbidities, from the correlation between (severe) infections and dementia incidence. The study included over 370,000 demographic-matched individuals aged 65 years or older in Finland, and adjusted rate ratio analysis suggests cystitis and bacterial infections of an unspecified site as valid risk factors for dementia independent of 27 recorded comorbidities, over 20 years of follow-up.**

**Strengths of the study include the size and comprehensiveness of the population, which includes all known individuals with dementia in a national cohort, over two decades. Some issues might be considered:**

**RESPONSE:** We agree with the reviewer about the general assessment of the study.

**POINT:**

**1. In the Methods section, it is stated that all individuals in the Finnish population aged 65+ at end-2016 and who were diagnosed with late-onset dementia between 2017 and 2020, were included; up to 5 controls were then sampled for each case. It might first be clarified as to whether a sufficiently large exposure window (up to 21 years before diagnosis/index date, i.e. from 1987) was available from register data, for all these individuals (e.g. recent immigrants). If not, were such individuals excluded from the analysis, or included with adjustments?**

**RESPONSE:** We agree that clarification on these details is necessary. All the 375,327 participants, including 62,555 dementia cases and 312,772 controls, resided in Finland both at the start of the exposure window (i.e. at the end of 1995–1998) and at the end of 2016. There were 525 dementia cases among recent immigrants who resided in Finland at end-2016 but not in 1995–1998. These were excluded from the analyses. Similarly, all the controls were selected from the population from which the small number of recent immigrants had been excluded. We have clarified this in the revised methods as follows:

*“**This observational study includes individuals aged 65 or older living in Finland in 2016 who were diagnosed with late-onset dementia between 2017 and 2020 and who also resided in Finland at the start of the exposure period on 1 January 1996–1 January 1999 (residence recorded on 31 December 1995–31 December 1998). Immigrants who arrived after the baseline of 1996–1999 were excluded.”* P. 8, lines 125-129

**POINT:**

**2. The number of controls for each dementia case (up to 5) might be explained. How was the maximum number of matched controls determined (e.g. according to theory, estimated prevalence, etc.)? Why "up to 5" controls, instead of a fixed 5 controls, given that sampling was performed with replacement? Was exact matching required for the three attributes (year of birth, sex, follow-up period)?**

**RESPONSE:** We chose 5 controls per case, because adding controls beyond 5 generally provides little gain in statistical power (Hennessy S, et al. PMID: 9921965), but increases computational demand. With a total population sample, like in our case, feasible computation times are an issue. Although sampling was done with replacement, the replacement was performed after matching the five controls for each case. We aimed at exactly 5 control per case, but because we required exact matching for year of birth, sex and follow-up period, there were some cases for whom 5 controls could not be found. These instances were, however, extremely rare. We had 62,555 dementia cases and 312,772 controls in the analysis, a difference of only 3 controls (0.00096%) from the desired number of 5 controls per case (5*62,555 = 312,775). We have clarified this in the revised manuscript as follows:

*“**Using incidence-density sampling with replacement, we aimed to identify 5 controls for each dementia case, matching exactly for year of birth, sex and follow-up period. Controls were required to be at risk (i.e., dementia-free) at the time of the matched case’s dementia diagnosis (the index date) but could later become cases if diagnosed with dementia after that date. Five suitable controls were found for almost all cases, resulting in a final sample of 62,555 dementia cases and 312,772 controls.”* P. 8, lines 129-135

**POINT:**

**3. In the Study design and sampling of controls subsection, it is stated that subjects who died were censored from the risk pool. It might be clarified as to the procedure for subjects who left the health system (e.g. by emigration).**

**RESPONSE:** We analysed all those who resided in Finland at end-2016 and were alive at end-2020 as if they had remained residents of Finland until end-2020. This assumption is justified, because very few older adults emigrate from Finland. According to Statistics Finland, of the 1 149 975 individuals in the total population aged 65+ at end-2016, only 663 (0.058%) emigrated in 2017. In 2018-2020, the annual emigration rate for people aged 65+ ranged from 0.045% to 0.051%. We have clarified this in the revised manuscript:

*“**Emigration after 2016 was very rare* *(approximately 0.05% annually) [21].* *Therefore, we assumed that all study participants at the end of 2016 continued living in Finland until the end of 2020 unless they died.”* P. 8, lines 136-138

We acknowledge the possibility of missing participants who emigrated during the follow-up in the revised Discussion:

*“We assumed that all participants remained covered by the Finnish health care system throughout the observation period, thereby ignoring any bias related to individuals who emigrated from Finland. However, this bias is unlikely to be major, as emigration among Finnish citizens aged 65 years and older is rare—approximately 0.05% annually [21].”* P. 24, lines 497-499

Refs.

<https://pxdata.stat.fi/PxWeb/pxweb/en/StatFin/StatFin__muutl/statfin_muutl_pxt_11a7.px/table/tableViewLayout1/>

<https://pxdata.stat.fi/PxWeb/pxweb/en/StatFin/StatFin__vaerak/statfin_vaerak_pxt_11rc.px/>

**POINT:**

**4. Moreover, the coverage of the register data might be discussed further. Would relevant data be missing due to individuals seeking care from private sources?**

**RESPONSE:** We retrieved register data from the Finnish Care Register for Health Care. In Finland, reporting all hospital care episodes and associated diagnoses to the register is mandatory for both public and private hospitals, although due to Finland’s comprehensive public health care system, most hospital care in Finland is given in public hospitals. We have clarified this in the revised manuscript:

*“The Finnish Care Register for Health Care covers both public and private health care and includes over 95% of all hospital discharges in Finland with high positive predictive values ranging from 75% to 99% for common diagnoses.^63^”* P. 24, lines 493-495

**POINT:**

**5. In the Hospitalisations from severe infections or other non-dementia illnesses (exposures) subsection, it is stated that non-disease events were not included in the analysis. Is it thus assumed that such events are uncorrelated to dementia incidence, despite some of them likely to be traumatic and/or chronic? This might be discussed.**

**RESPONSE:** Our definition of “disease” was broad, including also traumatic events such as injuries and poisonings (Chapter XIX in ICD-10). However, we agree that some of the non-disease events excluded from our study might be correlated with the incidence of dementia. We have discussed this in the revised Discussion of the manuscript as follows:

*“**Non-disease events, such as symptoms without a specific diagnosis, were outside the scope of the study, but might still contribute to dementia risk.^48^”* P. 25, lines 513-515

**POINT:**

**6. In the Ascertainment of all-cause dementia cases subsection, both ICD-10 and ICD-9 codes (used prior to 1996) are considered in defining prevalent dementia cases for exclusion. This appears to imply that this exclusion criteria extends to before the two-decade exclusion window. Is there a reason for the differing temporal criteria for comorbidities (up to 1996 only) and prevalent dementia (apparently up to 1987)? This might be briefly clarified.**

**RESPONSE:** In Finland, the use of ICD-9 started on 1 Jan 1987 and the use of ICD-10 on 1 Jan 1996. We looked for comorbidities 21-years backwards from dementia diagnosis, which, depending on the year of the dementia diagnosis (2017-2020), started on 1 Jan 1996 to 1 Jan 1999. This was the longest available time window for comorbidities, because, as mentioned above, the use of ICD-10 started in Finland on 1 January 1996. Looking beyond that was not possible, because we focussed on all disease-related ICD-10 codes and there is not always 1:1 correspondence between the codes of ICD-9 and ICD-10. Fortunately, for dementias, it is possible to look up all related ICD-9-codes even though the codes do not always match exactly. Thus, we used both ICD-9-codes (1 Jan 1987 to 31 Dec 1995) and ICD-10-codes (1 Jan 1996 onwards) to exclude prevalent dementia cases during and before the time window for comorbidities to make sure that all comorbidities were diagnosed before rather than after the dementia diagnosis. We have clarified this in the revised methods as follows:

*“Incident cases of dementia were recorded between 1 January 2017 and 31 December 2020. Participants with a record of prevalent all-cause dementia between 1 January 1996 (the inception of ICD-10 in Finland) and 31 December 2016 were excluded. To further ensure that the participants did not have prevalent dementia at the start of the exposure window (from 1 January 1996 to 1 January 1999), we also excluded participants who had a record of any dementia-related 9th revision (ICD-9) codes (290, 2900A, 2941A, 3310A, 3311A, 3312X, 3318X, 4378A) between 1 January 1987 and 31 December 1995, the time-period when ICD-9 was in use in Finland.”* P. 10, lines 169-176

**POINT:**

**7. In the Covariates subsection, it is stated that "to account for potential regional differences in health care, we adjusted analyses for area of residence, split into 18 regions". It might be clarified as to how these adjustments were made.**

**RESPONSE:** We agree and have clarified this in the revised Methods:

*“In Finland, public health care covers a great majority of hospitalisations and is organised on a regional basis. To account for potential regional differences in health care, we adjusted our models for area of residence using 19 dummy variables which corresponded to the administrative regional division confirmed by the Finnish Government. The three largest regions by population were Uusimaa (comprising the capital Helsinki and surrounding areas), Pirkanmaa (Tampere and surrounding areas) and Southwest Finland (Turku and surrounding areas).”* P. 11, lines 193-202

**POINT:**

**8. In the Statistical analysis subsection, it is stated that only diseases that preceded dementia with a prevalence of at least 1% during the exposure window were analyzed. Was the 1% threshold defined for achieving statistical significance on the cohort size, or other reasons? This might be clarified.**

**RESPONSE:** The 1 % threshold is analogous to the prevalence threshold for rare variants often used in population genetics (e.g., Momozawa Y, et al. J Hum Genet. 2021. PMID: 32948841, and Pathan, N et al. Nat Commun. 2024 Feb 9;15(1):1245. PMID: 38336875). We chose the 1% prevalence threshold to identify diseases that have potential public health relevance as risk factors for dementia. Although rare diseases might be associated with an increased risk of dementia, they are unlikely to have a major contribution to dementia on the population level. Additionally, including potentially hundreds or thousands rare conditions would have made our findings hard to interpret. We have clarified this in the revised methods as follows:

*“As in genetic studies of common versus rare variants, we chose the 1% threshold to focus on relatively common diseases that have relevance on the population health level.^25^”* P. 12, lines 208-210

**POINT:**

**9. In the Statistical analysis subsection, it is stated that in analyzing associations between dementia-related diseases, only the more commonly occurring temporal order was considered for each disease pair. This might be explained further - would not analyzing both orders be more complete, especially since there does not seem a requirement that causality between diseases is unidirectional?**

**RESPONSE:** As suggested by the reviewer, we conducted analyses of the less commonly occurring temporal order and summarise the findings in the revised manuscript. These analyses did not affect the interpretation of the associations between infections and dementia, because our original sensitivity analyses had already been adjusted for all dementia-related diseases. We explain this below in our next response to Point #10.

Methods:

*“**Thus, for each disease pair, we examined the more commonly occurring temporal order in primary analyses and the less common temporal order in supplementary analyses. “*P. 12, lines 225-227

Results:

*“In supplementary analyses examining the less commonly occurring temporal order, 79 (19.5%) of the 406 pairs remained significant (RR ≥ 1.20, p<0.00012) (**Table C in S1 Appendix).”* P. 17, lines 321-323

**POINT:**

**10. In the Statistical analysis subsection, from what could be understood, significant disease pairs were used to construct disease trajectories, before associations of diseases that were infections with dementia, were additionally adjusted for any other dementia-related diseases. Does the analysis of the latter (infections with dementia) rely on the previous analysis of disease pairs? In general, the statistical methodology should be described in greater detail, possibly in supplementary material.**

**RESPONSE:** To minimise the risk of residual confounding from dementia-related diseases, we adjusted the infection-dementia analyses for all dementia-related diseases regardless of whether they were identified in the disease-trajectory analysis. In the revised manuscript, we clarify this in the statistical methods section:

***“…****,* *we repeated the analyses on the associations between infections and dementia additionally adjusting for all other dementia-related diseases. To minimise the risk of residual confounding, these additional adjustments included all non-infectious dementia-related diseases regardless of whether they were components of the disease-trajectories leading to infections.”* P. 13, lines 240-244

**RESPONSE TO REVIEWER COMMENTS – REVIEWER #2:**

**POINT:**

**Reviewer #2: The authors investigated the role of comorbidities on the association between severe infections and dementia. This paper contributes to the growing literature on infections as a risk factor for dementia. Below are a few comments to be addressed.**

**1.	While the exposure period was 20 years in this study, it would be informative to know what the median or mean follow up was in this study.**

**RESPONSE:** The mean time from infection to dementia was 5.6 to 6.5 years depending on infection: 5.6 (SD 3.8) years for cystitis and 6.5 (SD 5.0) years for bacterial infection of unspecified site. We have highlighted this in the revised Results section:

*“The diseases were diagnosed on average 4.7 to 11.8 years before dementia diagnosis, with depression diagnosed the earliest (mean 11.8 [SD 6.2] years before dementia) and organic mental disorder the latest (mean 4.7 [SD 3.8] years). Cystitis and bacterial infection of unspecified site occurred on average 6.5 (SD 5.0) years and 5.6 (SD 3.8) years before dementia, respectively.”* P. 16, lines 300-304

**POINT:**

**2.	Given dementia can take many years to develop, the authors could mention the potential for misclassification bias in the control group.**

**RESPONSE: We agree. We have mentioned this in the revised Discussion:**

*“Some dementia cases may have been misclassified as controls owing to delayed dementia diagnosis; if present, this would likely attenuate the observed associations. Given the long preclinical phase of dementia, part of the observed associations might reflect preclinical dementia increasing susceptibility to infectious and other diseases, rather than the reverse, potentially leading to an overestimation of associations.“* P. 24, lines 503-507

**POINT:**

**3.	The authors could explain the rationale for the 1.20 RR cut off for selecting diseases for further analysis.**

**RESPONSE:** The 1.20 RR cut off for selecting diseases for further analysis was based on earlier studies that have considered it as the lower-bound for a small relative risk, relative risks <1.20 being very small (smaller than “small”) (Olivier J, et al. *Commun Stat* 2017; 46: 6774–81.; Kivimäki M, et al. Lancet Public Health. 2020 Mar;5(3):e140-e149.). Very small relative risks are more prone to bias and their public health relevance is also less certain (Siontis GC, et al. Int J Epidemiol. 2011 Oct;40(5):1292-307.). We have clarified this in the revised manuscript:

*“…, diseases showing a statistically significant association with dementia after Bonferroni correction (p<0.000294) and a RR of at least 1.20 were selected for further analysis. The RR threshold of 1.20 was chosen based on literature indicating that smaller effect sizes are often of limited public health relevance and more susceptible to bias, unless the disease incidence is very high.[15,28,29].”* P. 12, lines 214-218

**RESPONSE TO REVIEWER COMMENTS – REVIEWER #3:**

**POINT:**

**Reviewer #3:**

**1.	Abstract: The claim of "no loss to follow-up" is overstated. Patients may not re-enter the healthcare system or may receive diagnoses outside the captured registry system, leading to potential loss to follow-up.**

**RESPONSE:** We agree and have dropped this claim. Finland has a comprehensive population information system and the reporting of all hospital care episodes and associated diagnoses to the Finnish Care Register for Health Care is mandatory for both public and private hospitals. Additionally, due to Finland’s comprehensive public health care system, most hospital care in Finland is given in public hospitals. Further, very few older adults emigrate from Finland. According to statistics Finland, of the 1 149 975 total population aged 65+ at end-2016, only 663 (0.058%) emigrated in 2017. In 2018-2020, the annual emigration rate for people aged 65+ ranged from 0.045% to 0.051%. The relevant revised sections read:

Abstract:

*Background.* *Severe infections have been linked to an increased risk of dementia, but both conditions often coexist with other illnesses that may confound this association. Using nationwide Finnish health registry data, we examined the role of non-infectious mental and physical illnesses in the association between severe infections and dementia.* P. 2, lines 24-27

Methods:

*“Emigration after 2016 was very rare (approximately 0.05% annually) [21]. Therefore, we assumed that all study participants at the end of 2016 continued living in Finland until the end of 2020 unless they died.”* P. 8, lines 136-138

Discussion:

*“The Finnish Care Register for Health Care covers both public and private health care and includes over 95% of all hospital discharges in Finland”* P. 24, lines 493-494

*“We assumed that all participants remained covered by the Finnish health care system throughout the observation period, thereby ignoring any bias related to individuals who emigrated from Finland. However, this bias is unlikely to be major, as emigration among Finnish citizens aged 65 years and older is rare—approximately 0.05% annually [21].”* P. 24, lines 496-499

**POINT:**

**2.	The introduction lacks a clear statement of the study's specific aims and testable hypotheses. The research objectives and expected associations should be explicitly articulated.**

**RESPONSE:** We have revised the introduction as advised by the reviewer. The revised sections read:

*“**We anticipated identifying several infectious and non-infectious diseases that are interrelated and associated with dementia risk in Finnish adults. We hypothesised that severe infections increase the likelihood of developing dementia at least partly independently of preceding non-infectious comorbidities. Our specific aim was to assess the extent to which the association between severe infections and dementia risk remains after accounting for these conditions.“* P. 7, lines 100-105

**POINT:**

**3.	The authors describe this as a "prospective observational study," but the design is retrospective since it examines historical data from 2016-2020 to identify dementia cases diagnosed between 2017-2020.**

**RESPONSE:** We have omitted this description to avoid confusion. The relevant changes are as follows:

Title:

*“Severe infections and risk of dementia in Finland:* *A nationwide study of the role of non-infectious comorbidities”* P. 1, lines 4-5

Methods:

*”This observational study...”* P. 8, line 125

**POINT:**

**4.	The statement that controls "were also eligible to be included as cases if they had a diagnosis of dementia after the index date" raises concerns about potential misclassification bias due to delayed dementia diagnosis. How do the authors address controls who may have developed undiagnosed dementia during the study period? Especially those were choose as control cases but soon diagnosed with dementia.**

**RESPONSE:** We agree with the reviewer that participants chosen as controls but later diagnosed with dementia may artificially dilute associations. To address this concern, we conducted a sensitivity analysis in which we repeated the main analyses after excluding all controls who were diagnosed with dementia after the index date. The findings remained unchanged. For example, the RR for dementia associated with cystitis was 1.19 in both the original analysis and the sensitivity analysis. The corresponding RRs for dementia after bacterial infections of an unspecified site were 1.19 and 1.18, respectively. We report these analyses in the revised manuscript as follows:

Methods:

*“In sensitivity analyses, we additionally considered quintiles of household income as defined by Statistics Finland and medications for hypertension, diabetes and coronary heart disease based on medication reimbursement entitlements from the Social Insurance Institution of Finland.”* P. 11, lines 199-202

*“**We conducted several sensitivity analyses to test the robustness of our findings; we 1) included additional adjustments for income and prior medication use, 2) excluded all controls who developed dementia* *after the index date, 3) …”* P. 13, lines 246-249

Results:

*“The findings were consistent across sex and education strata (Figure 7) and in all sensitivity analyses (Figure A in S1 Appendix).”* P. 18, lines 347-349

**POINT:**

**5.	The choice of a "two-decade exposure window 1-21 years" (line 110) lacks justification. Why was this specific timeframe selected, and how does it relate to the natural history of dementia development?**

**RESPONSE:** The two-decade exposure window was selected for two reasons: First, dementia typically develops over a long period, often a decade or more (e.g., Amieva H, et al. Ann Neurol. 2008 Nov;64(5):492-8), so a sufficiently long exposure is required to capture relevant risk factors. Second, data availability constrained the time frame. In Finland, the ICD-10 was implemented on 1 January 1996, allowing a maximum observation window of 21 years (20-year exposure period plus a gap year). Although ICD-9 data exist for earlier years, a phenome-wide approach such as ours, covering all hospital-treated diseases, was not feasible with ICD-9 because of the lack of 1:1 correspondence between ICD-9 and ICD-10 codes. To ensure comprehensiveness and consistency, we therefore selected the longest available period based entirely on ICD-10 data.

We have clarified this in the revised manuscript as follows:

*“The exposure window was set at 21 years, corresponding the longest period for which ICD-10 data were available. A long exposure period was necessary because dementia typically develops over many years or even decades.^24^”* P. 9, lines 148-150

**POINT:**

**6.	While the authors control for demographic variables (sex, education, marital status, employment, residence), they omit potentially important confounders such as baseline cognitive scores, medication use, and socioeconomic status. This is particularly concerning given that the identified diseases may be associated with economic factors.**

**RESPONSE:** The reviewer suggests adjustments for a) cognitive scores, b) medication use and c) socioeconomic status. We respond to each of these suggestions:

a) We agree that scores from cognitive test batteries would be important, but as this study was based on registries covering the total Finnish population, such data were not available. It is important to appreciate the specific strengths of different study designs that can be used to complement each other. Cohort studies can be used to gather detailed baseline information from e.g. cognitive function, but they often suffer from issues with selective participation (typically those who are healthier are more likely to participate, e.g. Fry A, et al. Am J Epidemiol. 2017 Nov 1;186(9):1026-1034). By contrast, total population-based studies offer ample statistical power and fully represent the population better. In the revised manuscript, we have acknowledged the lack of cognitive scores as a limitation of the study as follows:

*“**As this is a population-wide register-based study, baseline cognitive assessments and clinical examination data leading to dementia and other disease diagnoses were not available. However, previous evidence indicates that electronic health record-based diagnoses have acceptable validity for examining disease risk factors.”* P. 24, lines 489-493

b) We have conducted analyses adjusted for medications for major chronic diseases: hypertension, coronary heart disease and diabetes. We report the corresponding statistical methods and findings, as described in the following point c.
c) The analyses in the originally submitted version were adjusted for education and employment, which reflect two relevant aspects of socioeconomic status. In the revised manuscript, we have added adjustment for quintiles of household income according to the official definition of Statistics Finland. We describe these data and statistical analyses and report the results in the revised manuscript as follows:

Methods:

*“In sensitivity analyses, we additionally considered quintiles of household income as defined by Statistics Finland and medications for hypertension, diabetes and coronary heart disease based on medication reimbursement entitlements from the Social Insurance Institution of Finland.”* P. 11, lines 199-202

*“**We conducted several sensitivity analyses to test the robustness of our findings; we 1) included additional adjustments for income and prior medication use, 2) excluded all controls who developed dementia after the index date, 3) used a year of birth, index year and sex-adjusted mixed effects logistic regression model with random effects for area of residence instead of the conditional logistic regression used in the main analyses 4) and used inverse-probability weighting to account for potential confounding by all the variables used in the most adjusted model (year of birth, index year, sex, education, marital status, employment, area of residence, income, medications for hypertension, diabetes and coronary heart disease and non-infectious dementia-related diseases).”* P. 13, lines 246-255

Results:

*“The findings were consistent across sex and education strata (Figure 7) and in all sensitivity analyses (Figure A in S1 Appendix).”* P. 18, lines 347-349

**POINT:**

**7.	The authors treat "area of residence, split into 18 regions" as fixed effects. Have they considered modeling regional variation as random effects, which might be more appropriate for geographic clustering?**

**RESPONSE:** We conducted sensitivity analyses in which we model regional variation using random effects instead of fixed effects. The results remained materially unchanged. For example, the RR for dementia associated with cystitis was 1.19 in the original analysis and 1.19 in the sensitivity analysis using random effects for regional variation. The corresponding RRs for dementia after bacterial infections of an unspecified site were 1.19 and 1.20, respectively. We report the methods and results of these sensitivity analyses in the revised manuscript as follows:

Methods:

*“We conducted several sensitivity analyses to test the robustness of our findings; we 1) included additional adjustments for income and prior medication use, 2) excluded all controls who developed dementia after the index date, 3) used a year of birth, index year and sex-adjusted mixed effects logistic regression model with random effects for area of residence instead of the conditional logistic regression used in the main analyses 4) and used inverse-probability weighting to account for potential confounding by all the variables used in the most adjusted model (year of birth, index year, sex, education, marital status, employment, area of residence, income, medications for hypertension, diabetes and coronary heart disease and non-infectious dementia-related diseases).”* P. 13, lines 246-255

Results:

*“The findings were consistent across sex and education strata (Figure 7) and in all sensitivity analyses (Figure A in S1 Appendix).”* P. 18, lines 347-349

**POINT:**

**8.	The choice of "at least 1.20 for dementia" as a clinically meaningful threshold (line 165) requires brief justification or reference to established criteria.**

**RESPONSE:** The 1.20 RR cut off for selecting diseases for further analysis was based on earlier studies that have considered it as the lower-bound for a small or moderate relative risk, the relative risk of <1.20 representing very small (smaller than “small”) (Olivier J, et al. *Commun Stat* 2017; 46: 6774–81.; Kivimäki M, et al. Lancet Public Health. 2020 Mar;5(3):e140-e149.). Although public health relevance also depends on the disease prevalence, very small relative risks are more prone to bias and their public health relevance is less certain (Siontis GC, et al. Int J Epidemiol. 2011 Oct;40(5):1292-307.). We have clarified this in the revised manuscript:

***“****…, diseases showing a statistically significant association with dementia after Bonferroni correction (p<0.000294) and a RR of at least 1.20 were selected for further analysis. The RR threshold of 1.20 was chosen based on literature indicating that smaller effect sizes are often of limited public health relevance and more susceptible to bias, unless the disease incidence is very high.^15,26,27^”* P. 12, lines 214-218

**POINT:**

**9.	The statement that "age and sex distributions were balanced between cases and controls" due to matching (lines 162-163) suggests these variables were not included in regression models. This is problematic since age and sex are potential confounders that should be controlled even in matched analyses.**

**RESPONSE:** We agree that age and sex are potential confounders that should be controlled even in matched analyses. We analysed the data using conditional logistic regression, which adjusts for all matching variables, because we used exact matching for sex and year of birth and because the odds ratios computed by conditional logistic regression are computed within matched groups (Wan F, et al. Am J Epidemiol. 2021;190:1859-1866, PMID: 33693492). However, as we pointed out in our response to point 7 of Reviewer #3 above, we additionally conducted sensitivity analyses using mixed effects models adjusted for age and sex; the results were consisted with those from the primary analysis. We have clarified these issues in the revised manuscript as follows:

*“…, after establishing the analytic design in the previous steps, we examined the strength of the associations between disease pairs using conditional logistic regression adjusted for education, marital status, employment, and area of residence. Adjustment for age and sex was inherent to the matched conditional design and analysis, as matching was performed on year of birth, index date and sex [27].”* P. 13, lines 229-233

*“We conducted several sensitivity analyses to test the robustness of our findings; we … 3) used a year of birth, index year and sex-adjusted mixed effects logistic regression model with random effects for area of residence instead of the conditional logistic regression used in the main analyses”* P. 13, lines 246-251

*“The findings were consistent across sex and education strata (Figure 7) and in all sensitivity analyses (Figure A in S1 Appendix).”* P. 18, lines 347-349

**POINT:**

**10.	The decision to examine only "the more commonly occurring temporal order" for disease pairs (lines 170-171) seems unnecessarily restrictive given the large sample size. Both temporal orders could be analyzed to provide more comprehensive insights.**

**RESPONSE:** This suggestion was made also by Reviewer #1, point 9. We conducted analyses of the less commonly occurring temporal order and summarise the findings in the revised manuscript. These analyses did not affect the interpretation of the associations between infections and dementia, because our original sensitivity analyses had already been adjusted for all dementia-related diseases.

Methods:

*“Thus, for each disease pair, we examined the more commonly occurring temporal order in primary analyses and the less common temporal order in supplementary analyses. “*P. 12, lines 225-227

***“…****, we repeated the analyses on the associations between infections and incident dementia additionally adjusting for any other dementia-related diseases. To minimise the risk of residual confounding, these additional adjustments included all non-infectious dementia-related diseases regardless of whether they were components of the disease-trajectories leading to infections”* P. 13, lines 240-244

Results:

*“In supplementary analyses examining the less commonly occurring temporal order, 79 (19.5%) of the 406 pairs remained significant (RR ≥ 1.20, p<0.00012) (Table C in S1 Appendix).”* P. 17, lines 321-323”

**POINT:**

**11.	The results in Figure 7 may be subject to selection bias. The authors should consider propensity score matching (PSM) or inverse probability weighting (IPW) to address potential confounding. The absence of economic status and other important covariates may bias the observed associations with infectious diseases.**

**RESPONSE:** To address potential confounding bias in the analyses presented in Figure 7, the reviewer suggests a) propensity score matching (PSM) or inverse probability weighting (IPW) and b) additional adjustments by economic status. We respond to both these concerns:

a) To address concern a), we have conducted sensitivity analyses using inverse probability weighting (IPW).
b) The analyses in the originally submitted version were adjusted for education and employment, which reflect two relevant aspects of socioeconomic status. To address concern b), in the revised manuscript, we have added adjustment for quintiles of household income according to the official definition of Statistics Finland.

We report these analyses and their results in the revised manuscript as follows:

*“In sensitivity analyses, we additionally considered quintiles of household income as defined by Statistics Finland and medications for hypertension, diabetes and coronary heart disease based on medication reimbursement entitlements from the Social Insurance Institution of Finland.”* P. 11, lines 199-202

*“We conducted several sensitivity analyses to test the robustness of our findings; we 1) included additional adjustments for income and prior medication use, 2) excluded all controls who developed dementia after the index date, 3) used a year of birth, index year and sex-adjusted mixed effects logistic regression model with random effects for area of residence instead of the conditional logistic regression used in the main analyses 4) and used inverse-probability weighting to account for potential confounding by all the variables used in the most adjusted model (year of birth, index year, sex, education, marital status, employment, area of residence, income, medications for hypertension, diabetes and coronary heart disease and non-infectious dementia-related diseases).”* P. 13, lines 246-255

Results:

*“The findings were consistent across sex and education strata (Figure 7) and in all sensitivity analyses (Figure A in S1 Appendix).”* P. 18, lines 347-349

**POINT:**

**12.	The entire section on "disease trajectories" and disease pairs is difficult to read and would benefit from clearer description is methods and presentation in results.**

**RESPONSE:** As suggested by the Reviewer, we have made extensive clarifications to the "disease trajectories" sections in the methods and results. The revised sections read as follows:

Methods:

*“In the first part of the data analysis, we identified diseases that were associated with an increased risk of incident dementia. This was done through three steps. First, we identified all diseases that preceded dementia with a prevalence of at least 1% during the exposure window (1–21 years before dementia diagnosis). As in genetic studies of common versus rare variants, we chose the 1% threshold to focus on non-rare diseases that have relevance on the population health level.26 Second, we analysed the associations between all these diseases and incident dementia using conditional logistic regression, adjusting for education, marital status, employment, and area of residence.* *Adjustment for age and sex was inherent to the matched conditional design and analysis, as matching was performed on birth, index date and sex.29 Third, diseases showing a statistically significant association with dementia after Bonferroni correction (P < 0.000294) and a RR of at least 1.20 were selected for further analysis. The RR threshold of 1.20 was chosen based on literature indicating that smaller effect sizes are often of limited public health relevance and more susceptible to bias, unless the disease incidence is very high.15,27,28*

*In the second part of the data analysis, we constructed disease trajectories across dementia-related diseases. This was done in four steps. First, we identified associations between pairs of dementia-related diseases among the dementia cases using the same 20-year exposure window as with the dementia analysis (from 1 January 1996–1 January 1999 to 31 December 2015–31 December 2018). Within each disease pair, the diseases can occur in two possible temporal orders (disease A before disease B or disease B before disease A). Thus, for each disease pair, we examined the more commonly occurring temporal order in primary analyses and the less common temporal order in supplementary analyses. Second, as in the analysis for incident dementia, we used incidence-density sampling for each disease, identifying up to 5 controls per disease case, matched for year of birth, index date and sex.* *Third, after establishing the analytic design in the previous steps we examined the strength of the associations between disease pairs using conditional logistic regression adjusted for education, marital status, employment, and area of residence. Adjustment for age and sex was inherent to the matched conditional design, as matching was performed on year of birth, index date and sex.29 P-values were adjusted for multiple testing using Bonferroni correction. Fourth, Bonferroni significant disease pairs in which the first disease had a RR of ≥1.20 for the occurrence of the second disease were retained in analysis. From these interconnected, dementia-related disease pairs, we constructed disease trajectories in which diseases were represented as nodes and the links between them as directed edges [15].*

*In the third part of the data analysis, we repeated the analyses on the associations between infections and incident dementia additionally adjusting for any other dementia-related diseases. To minimise the risk of residual confounding, these additional adjustments included all non-infectious dementia-related diseases regardless of whether they were components of the disease-trajectories leading to infections. We also assessed the associations of infections with dementia in subgroups defined by sex and education (tertiary or secondary [high] versus basic or no qualifications [low]). We conducted several sensitivity analyses to test the robustness of our findings; we 1) included additional adjustments for income and medication use, 2) excluded all controls who developed dementia after the index date, 3) used a year of birth, index year and sex-adjusted mixed effect logistic regression model with random effects for area of residence instead of the conditional logistic regression used in main analyses 4) and used inverse-probability weighting to account for potential confounding by all the variables used in the most adjusted model (year of birth, sex, education, marital status, employment, area of residence, income, medications for hypertension, diabetes and coronary heart disease and non-infectious dementia-related diseases).”* P. 12, lines 205-255

Results:

*“**In the first part of the analysis, we identified diseases robustly associated with incident dementia.* *Of the 170 diseases that preceded dementia with a prevalence of at least 1% during the 1–21-year exposure window, 59 showed statistically significant associations after Bonferroni correction for 170 tests (p<0.000294, Figure 2).* *Of these, 29 had a RR of at least 1.20 for dementia (Figure 3).* *These included 9 injuries, 6 mental and behavioural disorders, 5 cardiovascular and 3 neurological diseases, 1 endocrine, 1 metabolic, 1 eye and 1 digestive disease, as well as 2 infections (cystitis [an infection of the genitourinary system] and bacterial infection of unspecified site). Overall, 29,376 (47.0%) of the 62,555 dementia cases had at least one of these diseases during the two-decade exposure period before dementia, and 12,896 (20.6%) had at least two (Table A in S1 Appendix). The most common were cerebral infarction (prevalence 9.6%), intracranial injury (6.0%), and type 2 diabetes mellitus (4.9%). Numbers of dementia cases and controls with infections by type of infection are shown in Table 2.*

*The strongest associations with dementia were seen for mental disorders due to brain damage or physical disease (RR 3.76, 95% CI 3.48–4.06), Parkinson’s disease (RR 3.24, 95% CI 3.06–3.44), and alcohol-related mental and behavioural disorders (RR 1.87, 95% CI 1.76–1.99)(Figure 3).* *The RRs between infections and dementia were lower: 1.22 (95% CI 1.17–1.27) for cystitis and 1.21 (1.16–1.28) for bacterial infection of unspecified site. The diseases were diagnosed on average 4.7 to 11.8 years before dementia diagnosis, with depression diagnosed the earliest (mean 11.8 [SD 6.2] years before dementia) and organic mental disorder the latest (mean 4.7 [SD 3.8] years).* *Cystitis and bacterial infection of unspecified site occurred on average 6.5 (SD 5.0) years and 5.6 (SD 3.8) years before dementia, respectively.*

*In the second part of the analysis, we constructed disease trajectories from these 29 dementia-related diseases, forming 406 disease pairs.* *Considering the more commonly occurring temporal order in each disease pair, 153 pairs (37.7%) showed a RR ≥ 1.20 with a statistically significant p-value after Bonferroni correction (p<0.00012). These were used to construct a network of disease trajectories. Figure 4 presents trajectories with particularly strong associations (RR≥3.00); a 153 disease pairs are listed in the appendix (S2 Table). In supplementary analyses examining the less commonly occurring temporal order, 79 (19.5%) of the 406 pairs remained significant (RR ≥ 1.20, p<0.00012) (Table C in S1 Appendix).*

*The third part of the analysis examined whether the associations between infections and dementia were explained by other dementia-related diseases. Of the 29 dementia-related diseases identified in the first part of the analysis, 19 (65.5%) were associated with an increased risk of cystitis (Figure 5), with the strongest associations for epilepsy (RR 2.69, 95% CI 2.02–3.59), intracerebral haemorrhage (RR 2.69, 95% CI 1.88–3.85), and alcohol-related mental and behavioural disorders (RR 2.61, 95% CI 1.93–3.54). Ten diseases (34.5%) were associated with bacterial infections of unspecified site (mean 5.6 years (SD 3.8) before dementia diagnosis; Figure 6), most strongly other fluid, electrolyte and acid-base balance disorders(RR 2.57, 95% CI 1.95–3.40), retinal disorders in diseases classified elsewhere (RR 2.46, 95% CI 1.70–3.58), and alcohol-related mental and behavioural disorders (RR 2.30, 95% CI 1.77–2.99).*

*The associations of cystitis and bacterial infection of unspecified site with dementia risk remained robust after adjustment for all prior non-infectious dementia-related diseases (Figure 7). Only 10.8% to 13.8% of the excess dementia risk among individuals with these infections was attributable to comorbidities. The findings were consistent across sex and education strata (Figure 7) and in all sensitivity analyses (Figure A in S1 Appendix).”* P. 15, lines 282-349
