# Supplementary material for: The role of noninfectious comorbidities in the association between severe infections and risk of dementia in Finland: A nationwide registry study
Source: PLoS Med. 2026 Mar 24;23(3):e1004688. doi: 10.1371/journal.pmed.1004688 (PMC13012496; doi:10.1371/journal.pmed.1004688)
Supplement: Attachment — Submitted filename: Response_to_reviewers_R3.docx [file pmed.1004688.s008.docx]

-----------------------------------------------------------

Comments from the reviewers:

**RESPONSE TO REVIEWER #1:**

**POINT:** Reviewer #1: We thank the authors for addressing our previous comments, especially pertaining to temporal order between dementia-related diseases.

**OUR RESPONSE:** We thank the reviewer for their positive feedback.

**RESPONSE TO REVIEWER #4:**

**Reviewer #4:**

**POINT:** Summary: This nationwide study used Finnish health registry data with a case control approach to identify hospital-treated conditions associated with dementia in dementia cases and matched controls aged 65 years and over. Conditions coded with two infection-associated ICD-10 codes - cystitis and bacterial infection of unspecified site - as well as 27 non-infectious conditions were robustly associated with dementia after control for sociodemographic confounders. The two infectious diseases remained associated with RRs ~1.19 after adjustment for the 27 comorbidities. Associations were stronger for early-onset dementia and seen across a broader range of infections.

Originality and importance: There is increasing evidence that severe infections at a range of sites are associated with increased dementia risk. With the long trajectory of dementia development, it is uncertain whether these associations are causal and when in the course of dementia pathogenesis they act. This is an innovative and well-designed study that adds to the evidence that severe infections are likely to be independent dementia risk factors. It suggests that they act relatively late, potentially accelerating the onset of clinical dementia among those with pre-clinical or possibly prodromal dementia, rather than initiating neuropathological change. The study highlights the importance of prevention and rapid treatment of severe infections in older people to prevent cognitive and functional decline.

**OUR RESPONSE:** We thank the reviewer for an overview of our study and for their positive feedback.

Specific comments:

**POINT:** -	The aim stated at the end of the introduction was to assess the extent to which the association between severe infections and dementia risk remains present after accounting for preceding non-infectious comorbidities. It was not clear how the second part of the data analysis i.e. constructing disease trajectories across dementia-related diseases fit within that aim, especially when all non-infectious dementia-related diseases were adjusted for in the final analysis

**OUR RESPONSE:** Thank you for raising this issue. We believe the disease-trajectory analysis is an illustrative way to demonstrate the multitude of interconnections between dementia-related diseases. Our findings highlight the importance of taking non-infectious diseases into account when assessing associations between infections and dementia. We have clarified this in the revised introduction as follows:

*“We addressed this knowledge gap in the following ways. First, to ensure comprehensive analysis, we applied a phenome-wide approach, including all hospital presenting diseases or disease groups as defined in the International Classification of Diseases, 10th revision (ICD-10), with a prevalence of 1% or higher among dementia cases. We assessed which of these diseases were associated with an increased risk of dementia among dementia cases and controls drawn from the total population of Finland. Second, to examine the sequence, timing and inter-relatedness of the identified dementia-related diseases, we analysed disease trajectories and disease networks over 20 years preceding dementia onset, focusing on conditions that preceded both severe infections and dementia. Third, we assessed the extent to which the association between severe infections and dementia risk remains after accounting for other dementia-related diseases. We anticipated identifying several infectious and non-infectious diseases that are interrelated and associated with dementia risk in Finnish adults. We hypothesised that severe infections increase the likelihood of developing dementia at least partly independently of preceding non-infectious comorbidities.” P. 7, lines 98-112 (clean version)*

**POINT:** -	For the disease trajectory analysis, step 2 was to identify up to 5 matched controls per disease case. In the description of this process on p13, it was unclear whether every possible combination of disease pair was assessed i.e. was each ICD-10 code taken in turn as the disease? How successful was matching i.e. was it always possible to identify up to 5 individuals without the disease? Was the most common temporal order determined by the data or by any clinical input and a priori hypotheses?

**OUR RESPONSE:** We assessed each ICD-10 code in turn as the disease. It was not always possible to identify for all disease cases exactly 5 matched controls without the disease. The highest proportion of incomplete matches (0.4%) was for the 2959 cases with a Fracture of femur (ICD-10 S72). For 12 (0.4%) of these cases, the full number of 5 matched controls could not be identified. For each disease-pair, we determined the most common temporal order by the data without any clinical input of a priori hypotheses. However, the less common temporal order was additionally assessed and is presented in supplementary materials. We clarify these points in the revised manuscript as follows:

Methods:

*“In the second part of the data analysis, we constructed disease trajectories and networks across dementia-related diseases in four steps. First, we identified associations between all the possible pairs of dementia-related diseases identified in the first part of the data analysis. This was done among the dementia cases using the same 20-year exposure window as with the dementia analysis (from 1 January 1996–1 January 1999 to 31 December 2015–31 December 2018). Because any pair of diseases can occur in two temporal sequences (A→B or B→A), we first determined which sequence occurred more often in the data. That sequence was used in all primary trajectory analyses, while the alternative sequence was assessed in supplementary analyses. Second, as in the analysis for incident dementia, we used incidence-density sampling for each disease, identifying up to 5 controls per disease case, matched for year of birth, index date and sex. For virtually all disease cases, five appropriate controls were identified, with incomplete matches only occurring in ≤0.4% of cases.” P. 12, lines 228-240 (clean version)*

**POINT:** -	Clarify the meaning of 'directed edges' on p13 as it is not a term well-understood by clinicians

**OUR RESPONSE:** Directed edges is the same as arrows which is a more familiar term for the readers. We have therefore replaced directed edges with arrows in the revised version:

Methods:

*“From these dementia-related disease pairs, we constructed disease trajectories in which diseases were represented as nodes and their interconnections as arrows preserving the predominant temporal sequence [15]. For example, an arrow from depression to alcohol use disorder indicates that depression diagnosis more commonly preceded the diagnosis of alcohol use disorder in the data and that a diagnosis of depression was associated with an increased subsequent risk of alcohol use disorder.” P. 13, lines 246-252 (clean version)*

**POINT:** -	It was unclear how ICD-10 codes that dealt with similar/ the same conditions were considered. If investigated separately as if they were independent, would this lead to any limitations e.g. loss of power?

**OUR RESPONSE:** We considered each 3-digit ICD-10 separately regardless of whether there were any other 3-digit ICD-10 codes indicating an overlapping condition. For statistical significance, we used Bonferroni corrected p-values, which are considered conservative. The reviewer is correct that this may have caused us to miss some true associations. However, we would like to prefer to be conservative and minimise the risk of reporting chance findings. Additionally, because our study was large, a total of 62,555 dementia-cases and 312,772 dementia-free controls, statistical power remained high despite the quite conservative approach. In the revised methods section, we have clarified how the ICD-10 codes were treated. We also acknowledge the effects on statistical power in the revised limitations section of the Discussion.

Methods:

*“We analysed each 3-digit ICD-10 disease code as a separate entity and did not attempt to group codes that may refer to overlapping or clinically similar conditions” P. 9, lines 162-164 (clean version)*

Limitations section in Discussion:

*“We may have missed some true associations because we analysed each 3-digit diagnostic code separately, ignoring partial overlap between codes, and applied Bonferroni correction for multiple testing, which may be overly conservative when tests (i.e., diseases) are not fully independent.“ P. 25, lines 528-531 (clean version)*

**POINT:** -	In the limitations, you could note the lack of information on dementia subtypes. Previous studies have found stronger associations between severe infections and vascular dementia rather than Alzheimer's disease. It is possible that using an all-cause dementia outcome definition might have obscured some of these nuances

**OUR RESPONSE:** We agree and have acknowledged this limitation in the revised Discussion:

*“We examined all-cause dementia as the outcome. Future work should investigate dementia subtypes, given that severe infections may be more strongly linked to vascular dementia than to Alzheimer’s disease [1,3].” P. 25, lines 525-528 (clean version)*

**POINT:** -	The discussion did not consider why the early-onset dementia analysis identified additional infections compared to the main analysis

**OUR RESPONSE:** As suggested by the reviewer, we have discussed the differences in results between the analyses of early and late-onset dementia in the revised Discussion:

*“Consistent with previous findings, infections showed stronger associations with early onset than with late-onset dementia and we identified more dementia-associated infections in early onset dementia [4]. The mechanisms underlying these differences are unclear, but it is well-established that early and late-onset dementia differ in aetiology and genetic susceptibility [40,41].” P. 2, lines 3486-490 (clean version)*

**POINT:**-	On the study design diagram, figure 1, it would be helpful to show the covariate assessment window

**OUR RESPONSE:** As suggested by the reviewer, we have revised figure 1 to indicate the time of covariate assessment.

Any attachments provided with reviews can be seen via the following link:

--------------------------------------------------------- --- General editorial requests:

**RESPONSE TO GENERAL EDITORIAL REQUESTS:**

**POINT:** * Please confirm that your title complies with PLOS Medicine's style. Your title must be nondeclarative and not a question. It should begin with main concept if possible. "Effect of" should be used only if causality can be inferred, i.e., for an RCT. Please place the study design ("A randomized controlled trial," "A retrospective study," "A modelling study," etc.) in the subtitle (ie, after a colon).

**OUR RESPONSE:** The title complies with the PLOS Medicine’s style. The title is “Severe infections and risk of dementia in Finland: A nationwide study of the role of non-infectious comorbidities”

**POINT:** * Please confirm that your abstract complies with our requirements, including format (three sections: Background, Methods and Findings, and Conclusions) and providing all the information relevant to this study type https://journals.plos.org/plosmedicine/s/submission-guidelines#loc-abstract

**OUR RESPONSE:** The abstract is structured as instructed provides all the information relevant to this study type. To adhere to the submission guidelines, we have amended the abstract to describe the main limitations of the study:

Abstract Methods and Findings section:

*“We were not able directly to assess psychosocial, behavioural or biological confounders that are not captured in nationwide registries.” P. 3, lines 47-49 (clean version)*

**POINT:** * Please ensure that all abbreviations are defined at first use throughout the text.

**OUR RESPONSE:** We have checked that all abbreviations are defined at first use throughout the text.

**POINT:** * Please confirm that all numbers presented in the abstract are present and identical to numbers presented in the main manuscript text.

**OUR RESPONSE:** We checked this and found that some results that were reported in the abstract were presented in Figures but not in the manuscript main text. We have added them to the revised main text:

Results:

*“After these adjustments, the RR was 1.19 (95% CI [1.14, 1.24]; p<0.001) for cystitis and 1.19 (95% CI [1.13, 1.25]; p<0.001) for bacterial infections of unspecified site.“ P. 18, lines 360-362 (clean version)*

ABSTRACT

**POINT:** * In the abstract, please include the important dependent variables that are adjusted for in the analyses.

**OUR RESPONSE:** In the revised abstract, we describe these variables as follows:

*“Analyses were adjusted for education, marital status, employment and area of residence, with age and sex accounted for through the matched conditional design and analysis.“ P. 3, lines 31-33 (clean version)*

**POINT:** * In the last sentence of the Abstract Methods and Findings section, please describe the main limitation(s) of the study's methodology.

**OUR RESPONSE:** In the revised abstract, we describe the main limitation of the study methodology as follows:

*“We were not able directly to assess psychosocial, behavioural or biological confounders that are not captured in nationwide registries.” P. 3, lines 47-49 (clean version)*

AUTHOR SUMMARY

* In the author summary, in the final bullet point of 'What Do These Findings Mean?', please include the main limitations of the study in non-technical language.

**OUR RESPONSE:** We describe the main limitations of the study using non-technical language in the final bullet point of the revised author summary. We have also revised the final sentence of the Discussion better to contextualise our findings with the limitations of the study.

The final bullet point of the author summary:

*“Because this study is observational, intervention trials are required to establish whether better infection prevention could reduce dementia incidence.” P. 5, lines 76-77 (clean version)*

Concluding paragraph of Discussion:

*“Overall, our findings support the possibility that severe infections increase dementia risk; however, intervention studies are required to establish whether preventing or effectively treating infections yields benefits for dementia prevention.” P. 26, lines 550-552 (clean version)*

FIGURES AND TABLES

**POINT:** * When a p value is given, please specify the statistical test used to determine it in the legend.

**OUR RESPONSE:** We specify the statistical tests used to determine P-values in the legends of tables and figures as follows:

Legend for Table 2: *“…P-values were computed using the chi-squared test.” P. 35, line 762 (clean version)*

Legend for Figure 3: *“**Rate ratios and p-values are based on incidence-density sampling and matched analysis (conditional logistic regression) …” P. 17, lines 322-323 (clean version)*

Legend for Figure 4: *“…RRs and p-values are based on incidence-density sampling and matched analysis (conditional logistic regression) … RR, rate ratio.” P. 17, lines 342-343 (clean version)*

Legend for Figure 5: *“… RRs and p-values are based on incidence-density sampling and matched analysis (conditional logistic regression) … RR, rate ratio.” P. 19, lines 372-373 (clean version)*

Legend for Figure 6: *“… RRs and p-values are based on incidence-density sampling and matched analysis (conditional logistic regression) … RR, rate ratio.” P. 19, lines 382-383 (clean version)*

Legend for Figure 7: *“**Rate ratios and p-values are based on incidence-density sampling and matched analysis (conditional logistic regression) …” P. 19, lines 390-391 (clean version)*

Legend for Figure 8: *“Rate ratios and p-values are based on incidence-density sampling and matched analysis (conditional logistic regression) …” P. 21, lines 422-423 (clean version)*

Legend for Figure 9: *“Rate ratios and p-values are based on incidence-density sampling and matched analysis (conditional logistic regression) …” P. 21, lines 430-431 (clean version)*

**POINT:** * Please remove the P-values for the groups at baseline in Table 1.

**OUR RESPONSE:** We have removed the P-values for the groups at baseline in Table 1.

__________________________________________________

In compliance with data protection regulations, you may request that we remove your personal registration details at any time. (Use the following URL: https://www.editorialmanager.com/pmedicine/login.asp?a=r). Please contact the publication office if you have any questions.
