# Supplementary material for: The role of noninfectious comorbidities in the association between severe infections and risk of dementia in Finland: A nationwide registry study
Source: PLoS Med. 2026 Mar 24;23(3):e1004688. doi: 10.1371/journal.pmed.1004688 (PMC13012496; doi:10.1371/journal.pmed.1004688)
Supplement: Attachment — Submitted filename: Response_to_reviewers_R4.docx [file pmed.1004688.s009.docx]

**RESPONSE TO REVIEWER POINTS:**

Comments from Reviewers:

**POINT:** Reviewer #4: I am satisfied that my comments have been appropriately addressed in the revised version of this manuscript.

**OUR RESPONSE:** We thank the reviewer for their positive feedback.

Any attachments provided with reviews can be seen via the following link:

__________________________________________________

In compliance with data protection regulations, you may request that we remove your personal registration details at any time. (Use the following URL: https://www.editorialmanager.com/pmedicine/login.asp?a=r). Please contact the publication office if you have any questions.
